# Enhancing Patient Education with AI: A Readability Analysis of AI-Generated Versus American Academy of Ophthalmology Online Patient Education Materials

**DOI:** 10.3390/jcm14196968

**Published:** 2025-10-01

**Authors:** Allison Y. Kufta, Ali R. Djalilian

**Affiliations:** Department of Ophthalmology and Visual Sciences, University of Illinois Chicago, Chicago, IL 60612, USA

**Keywords:** artificial intelligence, patient readability, patient education, anterior segment, ophthalmology

## Abstract

**Background/Objectives**: Patient education materials (PEMs) in ophthalmology often exceed recommended readability levels, limiting accessibility for many patients. While organizations like the AAO provide relatively easy-to-read resources, topics remain limited, and other associations’ PEMs are too complex. AI chatbots could help clinicians create more comprehensive, accessible PEMs to improve patient understanding. This study aims to compare the readability of patient education materials (PEMs) written by the American Academy of Ophthalmology (AAO) with those generated by large language models (LLMs), including ChatGPT-4o, Microsoft Copilot, and Meta-Llama-3.1-70B-Instruct. **Methods**: LLMs were prompted to generate PEMs for 15 common diagnoses relating to cornea and anterior chamber, which was followed by a follow-up readability-optimized (FRO) prompt to reword the content at a 6th-grade reading level. The readability of these materials was evaluated using nine different readability analysis python libraries and compared to existing PEMs found on the AAO website. **Results**: For all 15 topics, ChatGPT, Copilot, and Llama successfully generated PEMs, though all exceeded the recommended 6th-grade reading level. While initially prompted ChatGPT, Copilot, and Llama outputs were 10.8, 12.2, and 13.2, respectively, FRO prompting significantly improved readability to 8.3 for ChatGPT, 11.2 for Copilot, and 9.3 for Llama (*p* < 0.001). While readability improved, AI-generated PEMs were on average, not statistically easier to read than AAO PEMs, which averaged an 8.0 Flesch–Kincaid Grade Level. **Conclusions**: Properly prompted AI chatbots can generate PEMs with improved readability, nearing the level of AAO materials. However, most outputs remain above the recommended 6th-grade reading level. A subjective analysis of a representative subtopic showed that compared to AAO, there was less nuance, especially in areas of clinical uncertainty. By creating a blueprint that can be utilized in human–AI hybrid workflows, AI chatbots show promise as tools for ophthalmologists to increase the availability of accessible PEMs in ophthalmology. Future work should include a detailed qualitative review by ophthalmologists using a validated tool (like DISCERN or PEMAT) to score accuracy, bias, and completeness alongside readability.

## 1. Introduction

Within ophthalmology, there are complex disorders and treatments associated with the cornea and anterior chamber. Ophthalmology has its own language and precise anatomy, which can be confusing for patients to understand. Clear and effective patient education materials (PEMs) are essential for enhancing understanding, supporting informed decision-making, and improving adherence to treatment plans. In ophthalmology, limited health literacy can directly contribute to medication and drop nonadherence and delayed recognition of urgent symptoms that signify potentially devastating disease processes or complications. Thus, improving readability of PEMs has direct implications for patient safety and vision outcomes. PEMs must prioritize accessibility, readability, and comprehension, ensuring that patients can fully benefit from the information provided and feel empowered in their own care.

The average American adult is estimated to read at an eighth-grade level, with 47% struggling to synthesize information from complex texts and nearly 20% unable to understand fourth-grade-level text [1]. Based on this, the American Medical Association and National Institute of Health recommend PEMs are below a 7th-grade reading level [2]. Organizations like the American Academy of Ophthalmology (AAO) and specialty-specific organizations have improved accessibility of high-quality online resources. While PEMs written by AAO are relatively easy to read, the range of topics is limited. PEMs published by other ophthalmology associations have been found to be too difficult to read [3]. For example, Eid et al. found that 16 brochures from the American Society of Ophthalmic Plastic and Reconstructive Surgery averaged an 11.2-grade reading level [4], while Cheng et al. [5] reported glaucoma PEMs at a 10.3-grade level, and pediatric ophthalmology online PEMs had an average grade level of 11.8 [6]. As such, there is a need for easily accessible patient education materials (PEMs) that cover a broader range of topics in ophthalmology, especially covering conditions affecting the cornea and anterior segment. AI chatbots may help clinicians optimize PEMs for better understanding.

In recent years, we have seen the rise of large language models (LLMs) and artificial intelligence (AI), and these have since been extensively explored as tools in healthcare. One interfacing format of these LLMs are chatbots, which allow the user to message the model in a conversational manner. After many years of development, OpenAI released ChatGPT (San Francisco, CA, USA, http://chatgpt.com) to the public in November 2022, with Microsoft following with Bing Chat (Copilot) (Redmond, WA, USA, https://copilot.microsoft.com) February 2023. Meta’s Meta-Llama, an open-source LLM, also debuted in February 2023 (Menlo Park, CA, USA, https://www.meta.ai/).

In ophthalmology, AI chatbots have been shown to be useful in assisting with charting, scientific writing, and are even capable of accurately answering board questions [7,8,9,10]. Through prompting, they are able to generate patient education materials (PEMs) on demand, though the quality and readability of AI-generated ophthalmic PEMs remain uncertain, as AI models can hallucinate.

Our study uniquely contributes to the field by being the first, to our knowledge, to evaluate the use of large language models (LLMs) in generating patient education materials (PEMs) specifically for corneal and anterior chamber diagnoses. Unlike previous research, we included open-source LLMs such as Meta-Llama-3.1-70B-Instruct, alongside ChatGPT-4o and Microsoft Copilot, thereby broadening the scope of AI tools assessed. 

This study aims to compare the readability of PEMs generated by ChatGPT4o, Microsoft Copilot, and Meta-Llama-3.1-70B-Instruct (utilized the software versions available online when accessed on 21 September 2024) with those on the AAO website, https://www.aao.org/eye-health/a-z (accessed on 7 January 2025) [11]. We hypothesize that by using prompt modifiers to adjust to a 6th-grade reading level, we can produce more readable PEMs than the existing ones. In addition to accessing readability, our goal is to explore the feasibility of integrating AI-generated and clinician edited PEMs into clinical workflows, where they could supplement existing resources, fill gaps in underserved topics, and be adapted in real-time to meet real-world needs.

## 2. Materials and Methods

The research was conducted in accordance with the principles of the Declaration of Helsinki, and no Health Insurance Portability and Accountability Act (HIPAA)-related information was used throughout the study. IRB/Ethics Committee ruled that approval was not required for this study.

The latest available editions of ChatGPT-4o, Microsoft Bing Chat/Copilot (Copilot), and the open-source Meta-Llama-3.1-70B-Instruct were prompted in late September 2024 on the following 15 common diagnoses relating to cornea and anterior chamber: corneal abrasion, cataract, keratitis, keratoconus, Fuchs dystrophy, conjunctivitis, corneal laceration, dry eye, iridocorneal endothelial syndrome, myopia, hyperopia, astigmatism, pinguecula and pterygium, scleritis, and trichiasis. ChatGPT’s temporary chat feature was utilized to discard any user memories and prevent the conversation from becoming training data. To emulate the temporary chat feature, a new chat was used each time in Copilot and Llama. To minimize confounding variables, web-search was disabled for all models.

This initial prompt was utilized as follows: act as a board-certified and fellowship-trained cornea, external diseases and refractive surgery ophthalmologist. For the following questions about an ophthalmic diagnosis, generate a 675-word patient education document (plus or minus 40 words) that is easily readable, comprehensible, and logical for patients of all educational backgrounds.

The topic is “[Topic]”.

The document must address the following questions:

1. What is the topic?
2. What are diagnostics involved, if any?
3. Why is treatment or procedure needed?
4. What are the risks and complications associated with the topic?

The target length of output was based on existing PEM materials from AAO. We picked AAO online PEMs due to their free, public availability and already high readability on several topics relevant to a cornea specialist.

Immediately after this, the following follow-up readability-optimized (FRO) prompt was used as follows: “Please reword your PEM output answer above at a 6th-grade reading level. Please try to keep word output as similar as possible”.

The outputs were saved as plain-text files and accessed using the following open-source program, cdimasci’s py-readability-metrics (https://github.com/cdimascio/py-readability-metrics accessed 22 September 2024) [12], which evaluates text though the following nine readability analysis libraries: Flesch–Kincaid Grade Level, Flesch Reading Ease Score, Dale–Chall Readability Score, Automated Readability Index (ARI), Coleman–Liau Index Grade Level, Gunning Fog Grade Level, Simple Measure of Gobbledygook (SMOG) Grade Level, Spache Readability Grade Level, and the Linsear Write Grade Level. An average of all Grade Level scores was used to create a combined grade level score. These libraries use various formulas based on factors like character and syllable numbers and sentence length.

### 2.1. Statistical Methods

**Figure 1:**
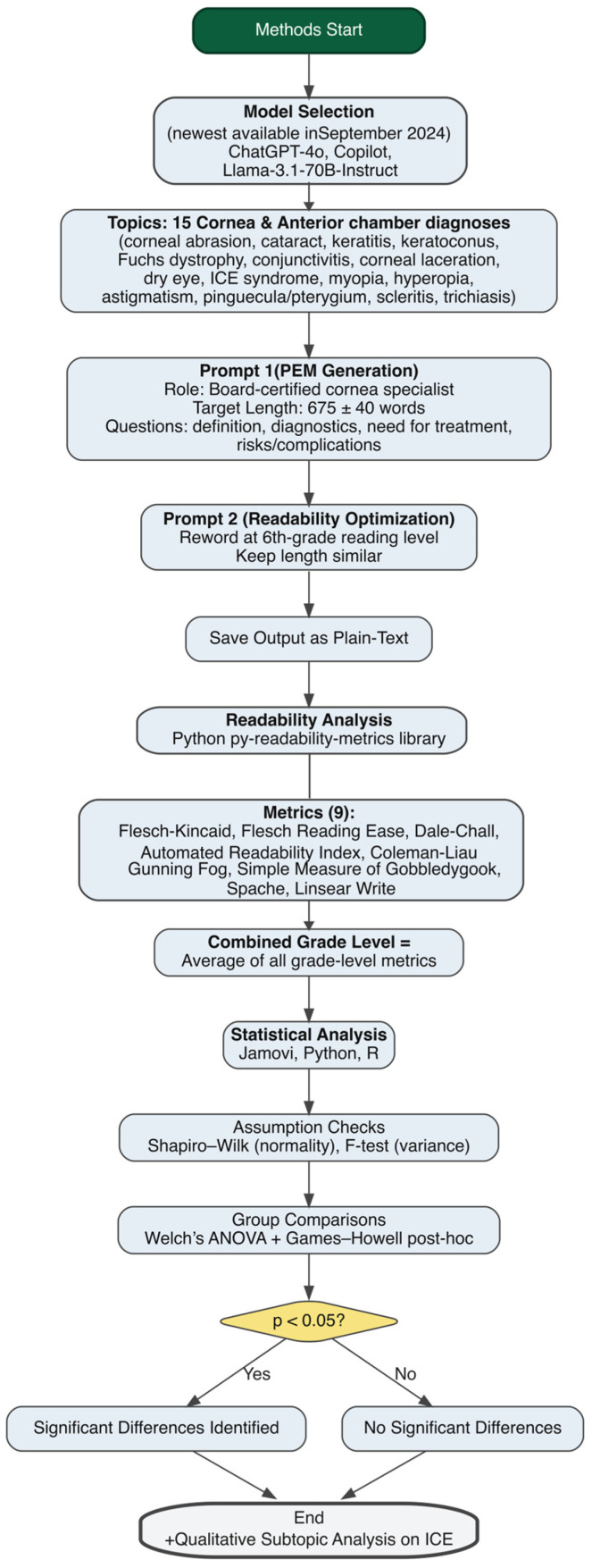
Methods flowchart.

Jamovi (Jamovi Project, Sydney, Australia, version 2.6.2 ), Python (Python Software Foundation, Beaverton, OR, USA, version 3.11.3), and R (The R Foundation, Online, version 4.5) were used to conduct statistical analyses as well as generate figures. The Shapiro–Wilk Test and F-test were used to analyze normality and equality of variance, respectively. Welch’s Analysis of Variance (ANOVA) was conducted with a Games–Howell (unequal variances) post hoc analysis to compare groups to each other and then to create pair wise comparisons, respectively. A threshold *p*-value of 0.05 was used to determine statistical significance. For a flowsheet detailing our methods, see Figure 1.

### 2.2. AI Assistance

During the preparation of this work, the authors used ChatGPT, Copilot, and Llama in order to create sample PEMs that were used in this analysis. Additionally, ChatGPT 4o was used in some areas to improve language and readability such as spelling and grammar. After using this service, the authors reviewed and edited the content as needed and take full responsibility for the content of the publication

## 3. Results

For all 15 topics, ChatGPT, Copilot, and Llama were able to create PEMs successfully based on the prompting process previously described.

Across all indices, the readability of all PEMs was generally at a higher grade level than the recommended 6th-grade level on average. The average of all grade-level readability indices for AAO PEMs was 8.0 ± 1.5 (Table 1).

**Figure 2:**
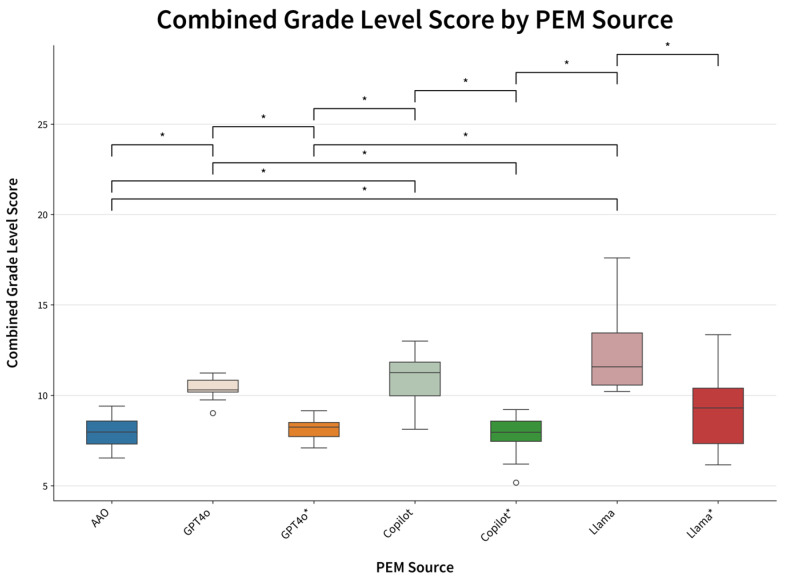
Comparison of combined grade level score by PEM source. * Denotes statistical significance with a *p*-value < 0.001.

**Figure 3:**
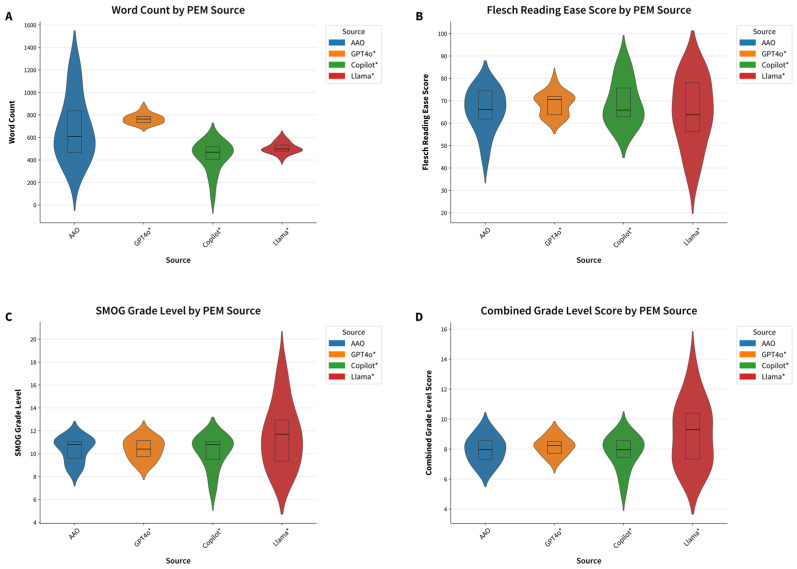
Comparison of AAO and follow-up readability-optimized prompted LLMs: (**A**) word count; (**B**) Flesch Reading Ease Score; (**C**) SMOG Grade Level; (**D**) combined grade level score. * In legend denotes usage of FRO prompt.

In comparison, initially prompted GPT4o, Copilot, and Llama had higher average readability grade levels of 10.8 ± 0.9, 12.2 ± 1.9, and 13.2 ± 3.1, respectively. When the FRO prompt was used to produce PEMs at a 6th-grade reading level, all AI models significantly improved their average readability grade levels: GPT4o to 8.3 ± 1.0, Copilot to 11.2 ± 1.9, and Llama to 9.3 ± 2.7 (*p* < 0.001 for all models comparing results of initial prompt and follow-up readability-optimized prompt is visualized in Figure 1). Similarly, word count improved after the readability-optimized prompt for all models, GPT4o 858 ± 30.4 to 766 ± 43.5, Copilot from 558 ± 68.6 to 428 ± 135, and Llama from 557 ± 39.9 to 507 ± 48.3 words, the latter two being significantly shorter than AAO materials at 681 ± 286 words (*p*-value 0.0006 and 0.034, respectively). However, while follow-up prompting significantly improved readability scores compared to the initial prompt, the AI-generated PEMs were not statistically easier to read than AAO materials (Figure 2 and Figure 3).

The initial outputs from all three AI chatbots had higher reading difficulty scores across all metrics compared to AAO materials, indicating that initially prompted AI-generated content was harder to read. Among the initial AI responses, Llama had the highest, most difficult-to-read Flesch–Kincaid Grade Level at 14.4 ± 5.0 and Flesch Reading Ease Score of 35.8 ± 16.0. In contrast, on average, GPT-4o had the lowest Flesch–Kincaid Grade Level at 10.9 ± 1.0 and the highest, more readable Flesch Reading Ease Score of 48.7 ± 6.9, but it still higher than AAO at 7.6 ± 1.4 and 65.7 ± 9.5, respectively.

A per topic, sub-analysis was also performed to further explore the data. FRO LLMs generated PEMs with word counts similar to AAO materials, though this varied by topic. It should be noted that Copilot outputs were much shorter in word count for most topics, even after FRO prompting (Supplementary Materials Figure S1A). Regarding word length, AAO alone had PEMs with over 1000 words for cataract, dry eye, and myopia.

The FRO prompts produced more readable PEMs in some topics when comparing Flesch Reading Ease Scores. See visualization in Supplementary Materials Figure S1B. A Flesch–Reading Ease Score > 80, indicating 6th-grade reading level, was achieved by Copilot for cataracts (87.5), corneal abrasion (85.6), and dry eye (81.0) as well as Llama for cataract (82.5), corneal abrasion (83.7), hyperopia (83.8), and myopia (81.7). For astigmatism, all LLMs had higher scores than AAO with FRO-prompted Copilot, Llama, and GPT4o with a reading ease of 74.8, 73.2, 72.6, and 64.5, respectively, as compared to AAO’s 64.5. However, AAO had a higher Flesch Reading Ease Score as compared to all LLM chatbots for conjunctivitis (66.1), Fuchs dystrophy (75.0), iridocorneal endothelial syndrome (76.9), and pinguecula and pterygium (70.0). See Supplementary Materials Figure S1B.

The other topics showed variance in the most readable PEM and the differences between topics were relatively minimal.

A qualitative comparison was performed between AAO and FRO LLM outputs for a representative topic, iridocorneal endothelial (ICE) syndrome. The AAO resource uniquely provided links to Supplementary Materials, a feature potentially reproducible by LLMs when web-search capability is enabled, although verification of source quality remains essential. This functionality was disabled during the present study to minimize confounding variables, preventing full application of the DISCERN criteria; however, several of its elements were incorporated into the subsequent analysis. Among the evaluated PEMs, AAO was the only source to acknowledge uncertainties and controversies in the pathogenesis of ICE, satisfying a key DISCERN criterion [13]. Both AAO and ChatGPT-4o addressed demographic risk factors, whereas only ChatGPT-4o delineated the three distinct ICE subtypes; in contrast, the AAO patient-facing article presented ICE as a collective group of disorders and this simplification may be easier to understand. ChatGPT-4o more explicitly linked diagnostic modalities to the underlying pathophysiology compared with Copilot and Llama, which provided only general descriptions of the tests. The AAO resource offered a concise explanation of expected ocular examination findings in plain language without naming specific tests. All FRO-prompted LLMs correctly indicated that management is directed toward the treatment of complications; however, only AAO explicitly emphasized that disease progression itself cannot be slowed by treatment. Copilot, the most brief of all PEMs, did not mention specific treatment strategies, but all other PEMs did. Llama uniquely discussed treatment-associated risks, thereby offering a more comprehensive risk–benefit perspective and its concision mainly from the use of bullet points instead of complete sentences.

These findings suggest that, with appropriate prompts and careful human review for nuance, accuracy, and completeness, AI language models can help physicians more quickly create PEMs that are comparable in length and readability to AAO content, which could aid in patient understanding. However, on average, both AI and AAO materials still exceeded the recommended 6th-grade reading level.

## 4. Discussion

This study indicates that prompted LLM AI chatbots can be a useful tool in creating easy-to-read patient PEMs. By prompting, we were able to generate PEMs with the LLMs that were at the 7–8th-grade reading level, much improved from the unprompted versions, but many topics were still higher than the NIH and AMA recommendation of 6th-grade. Even when directly prompted to write at below a 6th-grade reading level, the chatbots produced outputs on average that were above the 6th-grade reading level and did not outperform existing human-written materials (AAO). However, only one follow-up prompt was used, and more research is needed to explore prompt engineering to best optimize the creation of accurate and readable PEMs. It is of interest to investigate iterative prompting strategies and test differences in temperature or tone control, which may further enhance readability and preserve content integrity. Another potential investigation would be to directly incorporate the readability formulas as well as the DISCERN or PEMAT criteria. For example, iterative prompting (where prompts are refined across several rounds with feedback or clarification) has been shown to improve evidence recall, self-correction, and adaptability in LLM outputs, outperforming static prompting methods [14]. The of use chain-of-thought prompting to simplify complex terms could be also studied, though Jeon and Kim found that it did not significantly improve performance compared to simper approaches in medical question answering [15]. Advanced prompt engineering parameters should also be studied, as temperature can control randomness of the outputs. Specifically, lower temperatures (e.g., ≤0.3) produce more deterministic, predictable outputs while higher temperatures (e.g., ≥0.8) encourage creativity and linguistic variety but may reduce precision and factual fidelity [16]. Explicitly defining tone (e.g., empathetic, plain-language, narrative, etc.) represents another promising variable for future investigations, as instructing the model to adopt a conversational, empathetic, or instructive tone can influence readability and engagement without sacrificing content accuracy. Investigating whether specific tones (e.g., plain-language, narrative, or clinical) yield greater patient comprehension could inform best practices for tailoring PEMs to diverse literacy levels.

The average readability of AAO PEM was more readable than specialty-specific brochures assessed by similar studies, which showed PEMs having reading levels above 10th-grade. This investigation shows that prompted LLMs can be used to write readable PEMs that are on average around 7th-grade reading level when asked to output at a 6th-grade reading level. Given the average American is estimated to read at an eighth-grade reading level [1], integrating into a hybrid AI-ophthalmologist workflow could facilitate shared decision-making by increasing the number of available patient education materials, where patients better comprehend risks, benefits, and alternatives of surgical or medical interventions.

Performance varied by topic, and in some cases were more readable than AAO. Clinically, this means that AI may help reduce health literacy barriers for patients with anterior segment conditions where poor understanding of treatment adherence directly impacts outcomes. Given the limited number of topics that AAO’s website covers, AI could be a potential tool to assist in the creation of more PEMs, though physician review is *essential* to ensure accuracy and completeness. As our qualitative analysis shows, LLMs may omit nuance, particularly regarding areas of uncertainty or controversy. Nevertheless, ophthalmologists could employ AI as a structural scaffold to rapidly generate customized patient education materials with workflows that can be further customized to individual ophthalmologists and patients. For example, a human–AI hybrid workflow for pre- and postoperative care instructions, adjusted to the literacy level of the target population, has the potential to reduce severe complications such as endophthalmitis or corneal melt. Integration of such systems into electronic medical records could further streamline PEM creation, though physician editing and co-signature would remain essential to minimize harm and mitigate medicolegal liability from potential inaccuracies. Ethical considerations must be addressed, as certain tools lack source citations or may extract information out of context. Consequently, only audited and revised AI-generated materials should be used as final patient education resources due to the dynamic nature of model outputs and the aforementioned limitations.

A key strength of this study is its novel exploration of AI chatbots to enhance the readability of ophthalmology PEMs. However, limitations include the focus solely on readability rather than overall quality, such as content comprehensiveness and accuracy. Additionally, only one follow-up prompt was utilized, and variations in chatbot updates may affect reproducibility. The brevity of outputs from models such as Copilot raises concern for potential omission of essential content; however, this may reflect a stylistic preference for bullet points rather than full sentences rather than a true content deficiency, but a more comprehensive analysis is necessary. Additionally, readability algorithms may not fully capture advanced comprehension strategies like analogies or metaphors. Most importantly, human oversight and editing is instrumental as oversimplification may risk omitting important safety information.

The average readability of AAO PEMs in this study aligns with previous findings, showing they are more accessible than specialty-specific brochures, which often exceed a 10th-grade reading level. Unlike prior studies focusing solely on AAO materials, this investigation highlights the potential for LLMs to achieve comparable readability (though not at the 6th-grade gold standard) with broader customization and scalability. Such scalability is clinically relevant for various ophthalmic subspecialties, where existing PEMs are scarce, leaving patients with few reliable resources.

The higher-than-desired grade levels in AI-generated PEMs, even with direct prompting, may stem from limitations in prompt engineering and the intrinsic complexity of ophthalmologic topics. Despite these challenges, targeted prompting demonstrated the capability to improve readability and LLMs are helpful in brainstorming analogies, and these literary devices may enhance understanding in ways not measured by the readability analysis libraries. Additionally, AI models are becoming increasingly accessible to the public, with frequent models and upgrades. Variability in readability across topics suggests that AI-generated content could be optimized further with additional iterations or customized datasets. Clinicians could use this iterative refinement and prompt engineering to balance readability with accuracy, ensuring critical details (e.g., clinician specific nuances in clinical diagnosis and management, medication tapering schedules, and signs of urgent complications) are retained.

**Figure 4:**
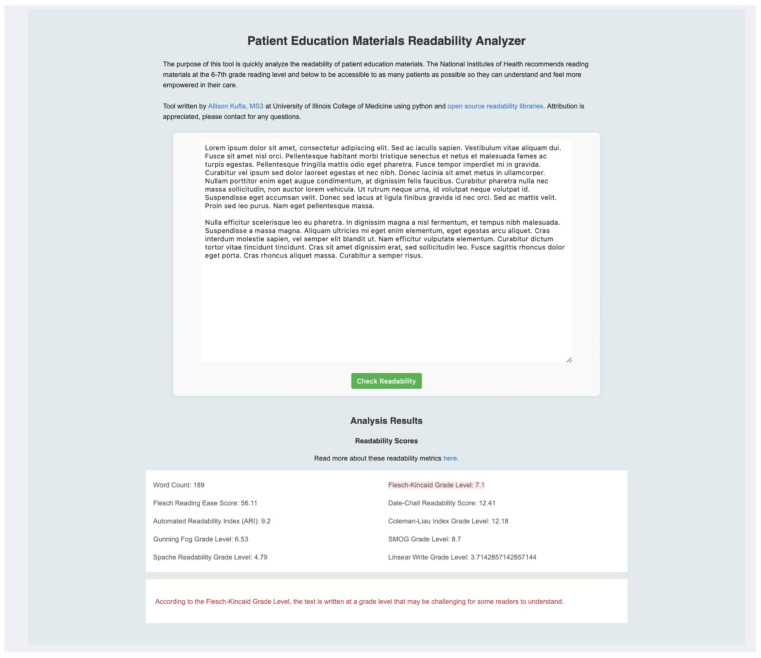
Screenshot of a simple program to evaluate any body of text for readability. Available at https://akufta.pythonanywhere.com.

Even with their limitations, AI chatbot tools are a great starting point for physicians to improve the number of accessible and readable PEMs for patients and careful prompting can reduce hallucinations, especially if instructed to use specific reference material only and cite sources properly. The authors have created a simple tool accessible at https://akufta.pythonanywhere.com (Chicago, IL, USA, Version 1, created 22 September 2024) to access readability of any body of text to make the creation and evaluation of PEMs easier (Figure 4). This tool could be integrated into clinical workflow, allowing physicians or other healthcare personnel to rapidly evaluate whether discharge instructions or informed consent documents meet literacy benchmarks before distribution.

Future research should evaluate AI-generated PEMs through subjective expert review and validated tools, such as DISCERN or PEMAT, to ensure both readability and quality. Exploring other models and how to correctly optimize prompts for readability would be of interest as well. Ultimately, artificial intelligence can be leveraged to more efficiently create PEMs that meet literacy standards, and therefore facilitate improved adherence, reduced anxiety, and increased patient safety.

AI chatbots show great potential for increasing the availability of easy-to-understand PEMs. While they are ever-evolving, can hallucinate, and their outputs require manual review, they can provide an excellent, efficient starting point for clinicians to create effective and accessible PEMs.

## Figures and Tables

**Table 1:** Patient education materials average readability metrics.

Measure	AAO	GPT4o	GPT4o *	Copilot	Copilot *	Llama	Llama *
Word count	681.0 ± 286.0	858.0 ± 30.4	766.0 ± 43.5	558.0 ± 68.6	428.0 ± 135.0	557.0 ± 39.9	507.0 ± 48.3
Flesch–Kincaid Grade Level	7.6 ± 1.4	10.9 ± 1.0	7.9 ± 0.8	11.6 ± 2.1	7.1 ± 1.8	14.4 ± 5.0	9.4 ± 4.1
Flesch Reading Ease Score	65.7 ± 9.5	48.7 ± 6.9	68.6 ± 5.1	40.8 ± 12.8	69.2 ± 10.1	35.8 ± 16.0	65.0 ± 15.1
Dale–Chall Readability Score	7.6 ± 0.6	8.4 ± 0.2	7.3 ± 0.2	9.2 ± 0.6	7.8 ± 0.5	8.9 ± 0.7	7.4 ± 0.7
Automated Readability Index	10.2 ± 3.0	13.8 ± 0.7	10.8 ± 0.8	14.8 ± 3.8	10.2 ± 1.5	17.9 ± 6.2	12.8 ± 4.9
Coleman–Liau Index Grade Level	11.3 ± 3.1	13.2 ± 0.9	10.0 ± 0.9	15.4 ± 3.6	10.2 ± 1.5	14.4 ± 1.6	10.1 ± 2.0
Gunning Fog Grade Level	8.0 ± 1.1	11.2 ± 0.5	8.8 ± 0.5	11.7 ± 1.5	8.1 ± 1.4	14.5 ± 4.8	10.5 ± 3.8
SMOG Grade Level	10.3 ± 1.1	13.3 ± 0.8	10.4 ± 0.9	13.4 ± 1.4	10.1 ± 1.5	15.8 ± 3.6	11.6 ± 2.8
Spache Readability Grade Level	4.1 ± 0.0	5.1 ± 0.0	4.3 ± 0.0	5.3 ± 0.0	4.1 ± 0.0	6.2 ± 0.0	4.9 ± 0.0
Linsear Write Grade Level	6.9 ± 0.4	10.9 ± 0.1	8.7 ± 0.2	10.0 ± 0.5	7.2 ± 0.4	14.0 ± 1.7	9.3 ± 1.4
Average Combined Grade Level	8.0 ± 1.5	10.8 ± 0.9	8.3 ± 1.0	11.2 ± 1.9	7.8 ± 1.4	13.2 ± 3.1	9.3 ± 2.7

*: AI-generated PEM with follow-up readability-optimized prompt to rewrite the initially generated content at a 6th-grade reading level. AAO, American Academy of Ophthalmology; SMOG, Simple Measure of Gobbledygook.

## Data Availability

Dataset available on request from the authors due to legal reasons regarding proprietary AI models, though raw AI outputs can be closely reproduced with prompts included in the paper. In addition to generating patient education materials, no other data was created. Data analysis libraries used are in the public domain, and the python package used is available at https://github.com/cdimascio/py-readability-metrics accessed 22 September 2024.

## Supplementary Materials

The following supporting information can be downloaded at: https://www.mdpi.com/article/10.3390/jcm14196968/s1, Figure S1: Comparison of LLMs by PEM Ophthalmology Topic and Source. (a) Word Count; (b) Flesch Reading Ease Score * denotes statistical significance *p*-value <0.001.

## Abbreviations

The following abbreviations are used in this manuscript:

PEMs	Patient Education Materials
LLM	Large Language Model
AAO	American Academy of Ophthalmology
SMOG	Simple Measure of Gobbledygook
AI	Artificial Intelligence
ANOVA	Analysis of Variance
